# Generation and characterization of a mouse line for monitoring translation in dopaminergic neurons

**DOI:** 10.1038/s41598-017-08618-2

**Published:** 2017-08-14

**Authors:** Joseph D. Dougherty

**Affiliations:** 1 Department of Genetics, Washington University School of Medicine, St. Louis, MO USA; 2 Department of Psychiatry, Washington University School of Medicine, St. Louis, MO USA

## Abstract

We developed a mouse line targeting midbrain dopamine neurons for Translating Ribosome Affinity Purification(TRAP). Here, we briefly report on the basic characterization of this mouse line including confirmation of expression of the transgene in midbrain dopamine neurons and validation of its effectiveness in capturing mRNA from these cells. We also report a translational profile of these neurons which may be of use to investigators studying the gene expression of these cells. Finally, we have provided the line to Jackson Laboratories for distribution and use in future studies.

## Introduction

Dopamine(DA) is an important neurotransmitter in the central nervous system. Though produced in limited cell populations throughout the mouse brain^1^, dopaminergic axons project widely throughout the nervous system, modulating a wide variety of circuits. Most notably, highly robust projections from the dopaminergic cells of the substantia nigra(SN) and ventral tegmental areas(VTA) to the striatum are essential for modulating behavior. Projections from VTA neurons to the nucleus accumbens have long been known to play a fundamental role in reward and are thought to be the common convergent pathway for all drugs of abuse^2^, while the dorsal-lateral striatum has a greater role in motor behavior^3^. Finally, both populations of neurons, but especially the SN, are vulnerable to genetic or environmental insults that result in their degeneration in patients with Parkinson’s disease. Thus, DA producing neurons have been a focus of intense scientific interest for decades with a deep cannon of accumulated knowledge about their morphology, projections, function, and physiology.

To enable study of translation specifically in DA producing cells of the mouse brain, we developed a transgenic mouse line expressing a ribosomal protein fused to GFP, eGFP/RPL10A, in DA neurons to permit Translating Ribosome Affinity Purification(TRAP) from these cells. Here we provide characterization of the expression of this line and validation of its ability to harvest mRNA from midbrain DA neurons. This line has now been distributed to Jackson laboratories(Stock# 030272) and should provide a resource for investigators interested in studying transcription and translation in these cells.

## Results

We generated two transgenic mouse lines to target this population of neurons. We first used a bacterial artificial chromosome(BAC) containing the tyrosine hydroxylase (*Th*) gene, a key enzyme in the synthesis of DA and norepinephrine which has traditionally been used as a marker of these cells, by replacing the coding sequence with the eGFP/RPL10a transgene. Characterization of eGFP expression in this mouse line showed some robust expression in the regions where DA neurons were known to be found, but also ectopic expression in TH negative populations in hypothalamus, striatum, and even sparse cells in cortex. There was also labeling in a subset of Purkinje neurons in the cerebellum, though this later pattern was somewhat consistent with some prior immunohistochemical data reporting TH expression in Purkinje cells^4^ and suggests that the enzyme has purposes in the CNS beyond the synthesis of DA and norepinephrine. It is also possible that this discrepancy reflects some level of difference between transcription and translation of the *Th* gene, and is consistent with a recent report of more widespread expression of Th mRNA than protein^5^. Although a pilot study demonstrated that TRAP could harvest RNA from midbrain dopaminergic cells(*data not shown)*, concerns about ectopic expression precluded further pursuit of this line. Thus, these first mice lacked specificity for DA producing neurons.

**Figure 1:**
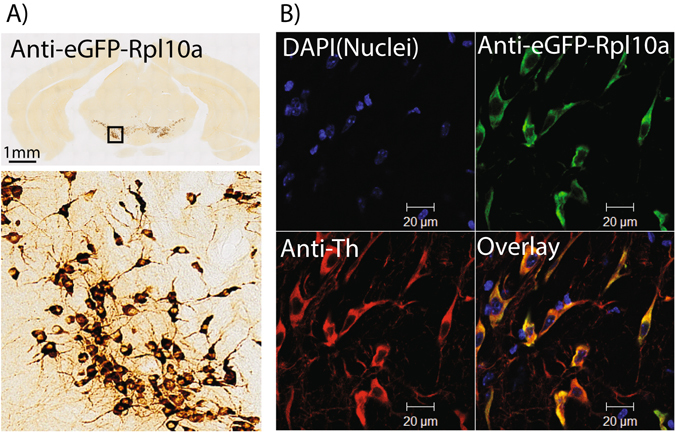
Anatomical confirmation of eGFP/RPL10A expression in midbrain dopamine neurons. (**A**) Anti-GFP immunohistochemistry shows regional expression of eGFP/RPL10A in neurons, consistent with expression in midbrain dopamine cells. (**B**) Immunofluorescence colocalization confirms expression in all Th positive neurons of the midbrain and only in Th positive neurons.

**Figure 2:**
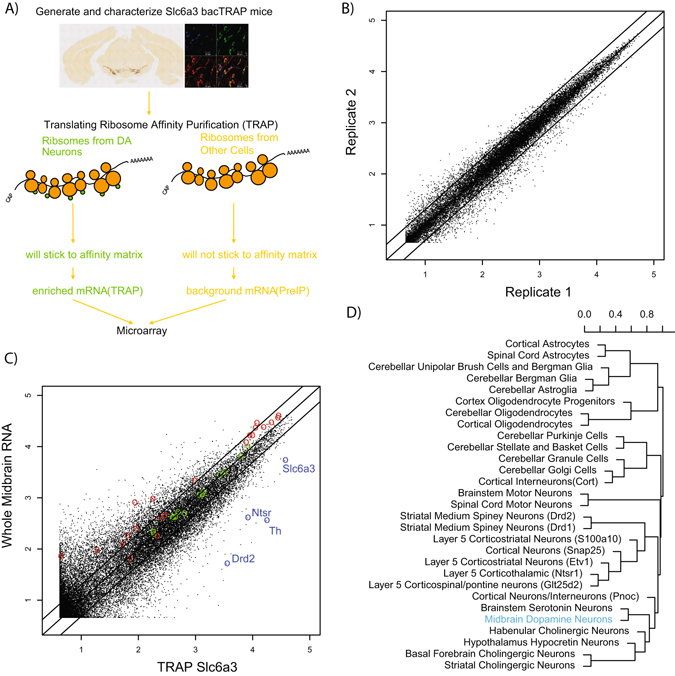
Slc6a3 JD1640 TRAP line allows for reproducible and specific purification or mRNAs from midbrain dopamine neurons. (**A**) Illustration of the bacTRAP method. Specific cell types are driven to express a GFP tagged ribosomal protein(RPL10A) using a cell type specific promoter in engineered from a bacterial artificial chromosome (BAC). After validation of the line, midbrains can be homogenized and lysates incubated with anti-GFP coated magnetic beads to enrich for mRNA from DA neurons. (**B**) Two microarray experiments on independent pools of Slc6a3 JD1640 TRAP mice result in reproducible mRNA expression (Pearson r > 0.98). (**C**) Comparison of TRAP purified from mRNA from Slc6a3 JD1640 TRAP to total RNA of midbrain shows robust enrichment of transcripts known to be expressed in dopaminergic neurons (blue), and moderate depletion of known glial genes (red). Lines in A and B show 2-fold enrichment or depletion (Pearson r > 0.90). (**D**) Hierarchical clustering of a variety of TRAP microarray datasets based on all transcripts with a pSI < 0.001 in any cell type reveals midbrain dopamine neurons(blue) are most similar to other neuromodulatory cells in enriched gene expression.

Therefore we next tested a BAC containing the *Slc6a3* gene, coding for the protein commonly known as the DA transporter(DAT). Immunohistochemical characterization of this mouse line revealed robust expression of eGFP/RPL10a in midbrain DA producing neuronal populations (Fig. 1A). Colabeling with TH antibodies revealed that TH positive neurons were consistently eGFP positive in these populations (Fig. 1B). We then isolated ribosome bound RNA from adult midbrain DA neurons and measured gene expression by microarray. Independent replicates showed high reproducibility (Fig. 2A). In addition, TRAP RNA was markedly different from parallel profiles of input RNA purified from the whole midbrain dissection (Fig. 2B). Specifically, a variety of known DA neuronal markers including Th (48 fold), Slc6a3 (6.8 fold), and Ntsr1(19.9 fold), were all significantly enriched in the TRAP sample (p < 0.003, p < 0.005, p < 0.05, respectively; LIMMA, with FDR correction). We believe the relatively lower enrichment of Slc6a3 likely represents saturation of the microarray probeset for this transcript in the TRAP sample, as there is no *a priori* reason to assume it should be substantially less enriched than Th, and the raw intensity values for the TRAP Slc6a3 probesets are in the top 0.05% of all probesets on the array. Non-neuronal ‘negative control’ transcripts were moderately depleted at a level typical of this TRAP protocol^6^.

**Figure 3:**
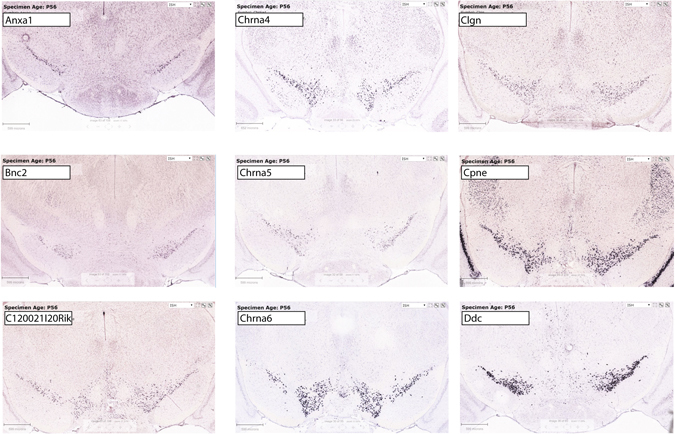
Examples of highly enriched transcripts in midbrain dopamine neurons. Shown are the first 9 genes of Table 1 with coronal *in situ* hybridization patterns available in the Allen Brain Atlas^25^. All show a regional pattern consistent with high and or/specific enrichment in midbrain dopamine neurons. Some (e.g. Anxa1) appear to only be expressed in a subset of the neurons, while others (Cpne) are highly expressed in SN/VTA but also show expression in additional populations. Images are from http://mouse.brain-map.org.

As a final validation of the new mouse reagent, we defined the set of most SN/VTA enriched transcripts (Table 1). Examination of a subset of these genes with publically available coronal *in situ* hybridization data confirmed high levels of expression for all in a pattern consistent with midbrain DA neurons (Fig. 3). Likewise, an analysis of enrichment of particular biological and molecular processes of the top enriched (pSI < 0.005) transcripts revealed enrichment of categories for “Ion Channel Activity” (p < 4.5E-5, Benjamini Hochberg corrected p-value), driven by transcripts such as *Kcnd3*, *Scn3a*, and *Chrna6*, “Dopamine Biosynthetic Process” (p < 1.3E-3), driven by transcripts for enzymes such as *Th*, *Gch*, *and Ddc* ^7^, and “Dopaminergic Neuron Differentiation” (p < 1.2E-2), driven by known master regulators such as *En1*, *Foxa2*, *Nr4a2*, *Pitx3*, and *Lmx1b* ^8^, consistent with identification of transcripts enriched in midbrain DA neurons.

**Table 1:** Probesets with >10 fold enrichment, p < 0.01, and pSI < 0.10e-6.

ProbeID	Mid	Slc6a3	FC	adj.P	pSI	Symbol	EntrezID	Long Name
1418601_at	7.9	2516.5	317.5	8.2E-04	1.9E-06	Aldh1a7	26358	aldehyde dehydrogenase family 1, subfamily A7
1448213_at	80.9	4191.1	51.8	9.6E-03	1.9E-06	Anxa1	16952	annexin A1
1438861_at	148.2	1517.3	10.2	3.2E-03	1.9E-06	Bnc2	242509	basonuclin 2
1455416_at	51.8	1630.8	31.5	4.1E-03	1.9E-06	C130021I20Rik	100504399	Riken cDNA C130021I20 gene
1446877_at	39.5	498.3	12.6	1.5E-02	1.9E-06	C230014O12Rik	329387	RIKEN cDNA C230014O12 gene
1421203_at	72.9	785.2	10.8	3.0E-02	1.9E-06	Chrna4	11438	cholinergic receptor, nicotinic, alpha polypeptide 4
1442035_at	7.4	309.3	41.6	3.3E-03	1.9E-06	Chrna5	110835	cholinergic receptor, nicotinic, alpha polypeptide 5
1450427_at	121.4	6196.6	51.1	5.2E-03	1.9E-06	Chrna6	11440	cholinergic receptor, nicotinic, alpha polypeptide 6
1418617_x_at	12.4	302.4	24.4	2.7E-03	1.9E-06	Clgn	12745	Calmegin
1433715_at	114.1	1516.6	13.3	4.4E-02	1.9E-06	Cpne7	102278	copine VII
1430591_at	4.6	82.1	17.9	4.5E-06	1.9E-06	Ddc	13195	dopa decarboxylase
1426215_at	2917.1	29921.0	10.3	1.3E-02	1.9E-06	Ddc	13195	dopa decarboxylase
1449939_s_at	18.5	1399.6	75.8	1.3E-02	1.9E-06	Dlk1	13386	delta-like 1 homolog (Drosophila)
1418618_at	12.8	1092.8	85.2	4.9E-03	1.9E-06	En1	13798	engrailed 1
1455872_at	24.3	3072.6	126.7	1.3E-02	1.9E-06	Fam167a	219148	family with sequence similarity 167, member A
1424695_at	4.7	314.3	67.5	3.2E-05	1.9E-06	Fam210b	67017	family with sequence similarity 210, member B
1421677_at	4.9	80.4	16.5	1.6E-02	1.9E-06	Fgf20	80857	fibroblast growth factor 20
1421855_at	23.2	289.9	12.5	2.9E-02	1.9E-06	Fgl2	14190	fibrinogen-like protein 2
1418496_at	239.6	6050.6	25.3	2.4E-02	1.9E-06	Foxa1	15375	forkhead box A1
1422833_at	25.7	705.7	27.5	1.2E-03	1.9E-06	Foxa2	15376	forkhead box A2
1441384_at	7.1	140.8	19.7	1.3E-03	1.9E-06	Gadl1	73748	glutamate decarboxylase-like 1
1429692_s_at	78.7	3291.0	41.8	1.4E-02	1.9E-06	Gch1	14528	GTP cyclohydrolase 1
1419593_at	5.8	91.7	15.8	7.3E-03	1.9E-06	Greb1	268527	gene regulated by estrogen in breast cancer protein
1436370_at	12.0	1766.4	147.8	7.0E-03	1.9E-06	Gucy2c	14917	guanylate cyclase 2c
1454783_at	12.1	737.0	60.8	1.0E-02	1.9E-06	Il13ra1	16164	interleukin 13 receptor, alpha 1
1436465_at	68.4	996.4	14.6	4.7E-02	1.9E-06	Klhl1	93688	kelch-like 1
1449241_at	102.2	1395.2	13.7	1.9E-02	1.9E-06	Klhl1	93688	kelch-like 1
1441729_at	68.7	846.1	12.3	1.1E-02	1.9E-06	Lmx1b	16917	LIM homeobox transcription factor 1 beta
1453225_at	6.2	120.9	19.5	6.9E-02	1.9E-06	Ndnf	68169	neuron-derived neurotrophic factor
1450750_a_at	145.4	3136.9	21.6	4.6E-03	1.9E-06	Nr4a2	18227	nuclear receptor subfamily 4, group A, member 2
1420799_at	403.7	8036.3	19.9	3.1E-02	1.9E-06	Ntsr1	18216	neurotensin receptor 1
1449917_at	4.6	195.7	42.6	3.5E-04	1.9E-06	Pitx3	18742	paired-like homeodomain transcription factor 3
1421359_at	4.6	427.4	93.2	1.2E-02	1.9E-06	Ret	19713	ret proto-oncogene
1436359_at	2072.3	24126.0	11.6	7.8E-03	1.9E-06	Ret	19713	ret proto-oncogene
1437079_at	322.1	11457.0	35.6	2.6E-02	1.9E-06	Slc18a2	214084	solute carrier family 18 (vesicular monoamine), member 2
1451139_at	4.6	190.1	41.1	9.8E-03	1.9E-06	Slc39a4	72027	solute carrier family 39 (zinc transporter), member 4
1455442_at	5.2	246.1	47.5	1.7E-02	1.9E-06	Slc6a19	74338	solute carrier family 6 (neurotransmitter transporter), member 19
1427919_at	13.1	347.2	26.6	1.0E-02	1.9E-06	Srpx2	68792	sushi-repeat-containing protein, X-linked 2
1437190_at	7.5	126.4	16.8	4.8E-03	1.9E-06	Styk1	243659	serine/threonine/tyrosine kinase 1
1449816_at	7.5	172.4	23.0	1.6E-05	1.9E-06	Sult5a1	57429	sulfotransferase family 5A, member 1
1422955_at	34.7	1163.7	33.6	3.2E-02	1.9E-06	Syt17	110058	synaptotagmin XVII
1437029_at	79.5	1777.9	22.4	5.0E-02	1.9E-06	Tacr3	21338	tachykinin receptor 3
1420546_at	356.1	17293.2	48.6	2.1E-03	1.9E-06	Th	21823	tyrosine hydroxylase
1445371_at	8.8	237.1	26.9	5.8E-03	1.9E-06	Tmem207	100043057	transmembrane protein 207
1449431_at	7.2	431.1	60.0	1.2E-05	1.9E-06	Trpc6	22068	transient receptor potential cation channel, subfamily C, member 6
1425163_at	8.2	184.6	22.5	9.4E-04	1.9E-06	AI661453	224833	expressed sequence AI661453
1460425_at	4.6	154.2	33.6	2.3E-04	1.9E-06	1700001C19Rik	75462	RIKEN cDNA 1700001C19 gene

*ProbeID*: Affymetrix Probe ID, *Mid*, *Slc6a3:* Expression level in midbrain Input RNA and Slc6a3 TRAP RNA, respectively. *FC:* ‘Fold-Change’ value of Slc6a3/Mid. *adj*.*P:* LIMMA FDR adjusted p-value for Slc6a3 vs Mid. *pSI*: Specificity index statistic p-value, when Slc6a3 is compared to other TRAP samples (Fig. 2C). *Symbol*: Official gene symbol. *EntrezID:*Entrez Gene ID. *Long Name:* Official gene name.

## Discussion

We report here the generation and characterization of a mouse line capable of translationally profiling midbrain dopamine-transporter expressing neurons. We show robust expression in all midbrain Th positive neurons, and confirm the ability of the mouse line to enable translational profiling. Thus, the mouse line should be useful to investigate ribosome bound transcripts in these neurons both at baseline, and in response to experimental manipulations, such as stimulation by drugs of abuse. We have also recently adapted a procedure for nuclear RNA purification from TRAP mouse lines^9^, so both nuclear transcription and cytoplasmic translation are in theory accessible using this line, and others have shown that the same basic approach can be applied to study the epigenetic profile of specific neuronal cell types^10^.

It is an interesting question as to why the Th bacTRAP line showed expression outside of TH positive cells. In the modified BAC designed to express eGFP/RPL10A in lieu of TH, the eGFP/RPL10a transgene sequence, followed by a strong polyA signal, is inserted at the translational start site of the *Th* gene. Thus, the transgene will co-opt the promoter/enhancers of the *Th* gene, due to the polyA signal but not the 3′ UTR. Thus, to the extent TH mRNA might be transcriptionally expressed, but translationally suppressed by 3′ UTR sequences in ‘ectopic’ populations, one would expect to detect eGFP/RPL10a protein in cells where TH protein is absent. Such translational regulation might also explain why many Th Cre lines, including knock-ins and transgenics, show recombination in Th negative cells^11^.

We also note that the new Slc6a3 TRAP line is distinct from the one recently used to successfully profile midbrain DA neurons in an MPTP Parkinson’s model^12^. Both lines were generated by modifying the same initial BAC, however the current line was initiated on the FVB strain and subsequently backcrossed to C57BL6/J mice, while the other line was directly generated on C57 mice from Charles River. Thus there will be modest strain differences between the two because of the different sources of C57 mice and any remaining introgressed FVB alleles in linkage with the transgene. Also, as they are separate integrants, they are expected to differ in transgene copy number and location. However, the line reported here is the exact line used to successfully profile embryonic midbrain DA neurons^5^. As the current line is being released by Jackson labs, we have provided this brief report to provide details of the generation and characterization of the line for future investigators, especially as minor differences between expected and actual strain or copy number could influence experimental results. Also, though they were generated independently of GENSAT, neuroanatomical data for this and other published bacTRAP lines are now also being hosted at the GENSAT website for the convenience of the field.

The translational profile of DA neurons provided here may also be of use to investigators interested in these cells. For example, it has recently been noted that midbrain DA neurons can co-release the neurotransmitter GABA^13^, yet do not appear to contain the traditional GABA synthesizing enzymes GAD65 and GAD67^14^ coded for by the genes *Gad1* and *Gad2*. Our microarray data is consistent with this expression pattern, showing robust depletion of these two genes from the TRAP sample (e.g. Gad1 6588 arbitrary expression units in the input RNA, and only 2396 in the TRAP, with this remaining TRAP signal likely reflecting non-specific background). However, the Gad paralog *GadL1* is 19 fold enriched in midbrain DA neurons (p < 0.002), though likely expressed only at low levels (absolute expression level: 140.8). Although normally thought to be involved in synthesis of Carnitine or Taurine rather than GABA^15, 16^, several unstudied splice isoforms are present in the UCSC genome browser. If any of the unstudied splice isoforms do produce GABA this might contribute to GABA neurotransmission from DA cells. Thus, to enable investigators to query our findings for additional insights, the complete analyzed data is provided as Supplemental Table 1.

## Methods

### Generation of mouse line

All procedures involving animals were approved by the Animal Studies Committee of Washington University in St. Louis and the Rockefeller University Institute Animal Care and Use Committee. All methods were carried out in accordance with relevant guidelines and regulations. A BAC RP24-269I17, was modified as described^17^, to insert the TRAP construct EGFP/RPL10A^18^ into the translation start site of the *Slc6a3* gene. Recombination was confirmed by Southern blot. Modified BAC DNA was purified by CsCl centrifugation and injected into fertilized FVB oocytes as described^19^. Founder mice were bred to C57BL6/j mice for subsequent generations. Mice were maintained as trans-heterozygotes and transgene carrying pups were identified at each generation by genotyping tail clip DNA for GFP.

### Translating Ribosome Affinity Purification

Five adult mice per replicate were euthanized with CO_2_, and midbrains were dissected in ice cold buffer in the presence of cyclohexamide to stall translation. TRAP was conducted as described^20^. Parallel input fractions were collected from each replicate as a measure of whole midbrain tissue RNA composition. RNA quantity and quality were determined with a Nanodrop 1000 spectrophotometer and Agilent 2100 Bioanalyzer with PicoChip reagents. All RINs were above 7. For each replicate, up to 10 ng of total RNA was amplified with the Affymetrix two-cycle amplification kit and hybridized to Affymetrix 430 2.0 microarrays according to the manufacturer’s instructions, and data were processed in R as described^6^, using GCRMA for normalization and identification of specific and enriched genes using the pSI package^6^ with default settings, compared to a large set of prior cell types analyzed by TRAP on the same microarray platform^19, 21–24^.

### Anatomical analysis

For immunohistochemistry(IHC), mice were euthanized then perfused transcardially with PBS, followed by PBS with 4% paraformaldehyde. Brains were dissected, cryoprocted with 30% sucrose in PBS then processed MultiBrain Technology (NSA, NeuroScience Associates, Knoxville, TN) for DAB IHC with a 1:75,000 dilution of Goat anti-EGFP serum according to the Vectastain elite protocol (Vector Labs, Burlingame, CA). Serial sections were digitized with a Zeiss Axioskop2 microscope at 10× magnification.

For immunofluorescent studies, brains were prepared as above, frozen and sectioned to 40 uM on a crytostat, and stored in PBS with 0.1% azide until use. Sections were blocked with 5% normal donkey serum and 0.25% triton and then incubated with Chicken anti-GFP(Abcam) and Mouse anti-Th (Chemicon) followed by appropriate Alexa dye-conjugated secondary antibodies (Invitrogen, Carlsbad, CA). Images were acquired as Z stacks (2 μm sections) with a Zeiss Inverted LSM 510 confocal microscope.

For Allen Brain Atlas images of Fig. 3, we selected for presentation the first 9 available coronal *in situ* hybridization image sets, alphabetically, from those transcripts with > 10 fold enrichment, p < 0.01, and pSI < 0.10e-6 (Table 1).

### Data Availability

Analyzed data are available as Supplemental Table 1. Raw data are available at GEO: GSE99927.

## Electronic supplementary material

Supplementary information

Dataset 1
