# Supplementary material for: Generation and characterization of a mouse line for monitoring translation in dopaminergic neurons
Source: Sci Rep. 2017 Aug 14;7:8117. doi: 10.1038/s41598-017-08618-2 (PMC5556054; doi:10.1038/s41598-017-08618-2)
Supplement: Supplementary file 1 — Supplementary information [file 41598_2017_8618_MOESM1_ESM.pdf]

*Supplemental information for*:
**Generation and characterization of a mouse line for monitoring translation in dopaminergic neurons.**

Joseph Dougherty[1,2]

[1]Department of Genetics, Washington University School of Medicine, St. Louis, MO, USA
[2]Department of Psychiatry, Washington University School of Medicine, St. Louis, MO, USA

**Contact:**
Dr. Joseph Dougherty
Department of Genetics
Campus Box 8232
4566 Scott Ave.
St. Louis, MO. 63110-1093
P: 314-286-0752
F: 314-362-7855
E:jdougherty@genetics.wustl.edu

**Running Title** (50 characters): **Monitoring translation in dopaminergic neurons**

**Supplemental Table 1: Analyzed data for all probesets.**

*ProbeID*: Affymetrix Probe ID, *Mid, Slc6a3:* Expression level in midbrain Input RNA and Slc6a3 TRAP RNA, respectively. *FC:* 'Fold-Change' value of Slc6a3/Mid. *adj.P:* LIMMA FDR adjusted p-value for Slc6a3 vs Mid. *pSI*: Specificity index statistic p-value, when Slc6a3 is compared to other TRAP samples (Figure 2C). *Symbol*: Official gene symbol. *EntrezID:*Entrez Gene ID. *Long Name:* Official gene name. Foldchange and pSI are only calculated for those transcripts determined to be above background as described[6].
